# Huntington's disease: A clinical primer for acute and general physicians

**DOI:** 10.1016/j.clinme.2024.100200

**Published:** 2024-04-06

**Authors:** Thomas H. Massey, Duncan J. McLauchlan

**Affiliations:** a University Hospital of Wales, Cardiff & Vale University Health Board, Cardiff, UK; b Centre for Neuropsychiatric Genetics and Genomics, School of Medicine, Cardiff University, Hadyn Ellis Building, Maindy Road, Cardiff, UK; c UK Dementia Research Institute at Cardiff University, Cardiff, UK; d Morriston Hospital, Swansea Bay University Health Board, Swansea, UK

## Abstract

Huntington's disease (HD) usually manifests in adulthood and is characterised by progressive neurodegeneration in the brain that causes worsening involuntary movements, mental health and cognition over many years. Depression, anxiety and apathy are common. HD is autosomal dominant and affects about 1 in 8,000 people in the UK. There are currently no disease-modifying treatments and so patient care centres on multidisciplinary therapy support and medical treatments to relieve distressing symptoms. Progression of HD is usually slow, and so acute deteriorations often indicate another problem, such as intercurrent infections, constipation, urinary retention, gastro-oesophageal reflux disease or poor dentition. In this review we outline common presentations in HD patients, both acute and chronic, consider therapeutic options and discuss specific considerations in advanced HD.

## Introduction

Huntington's disease (HD) is an autosomal dominant neurodegenerative disorder characterised clinically by progressive deterioration of movement control, mental health and cognition.^1^ Symptoms typically start in middle-age and progress relentlessly over the next 10–20 years, causing considerable morbidity. HD often leads to early loss of employment, reduced motivation and disordered behaviours. Over time, individuals lose the ability to function in daily life and become unable to care for themselves. HD reduces life-expectancy by an average of 13.3 years^2^ and there is currently no disease-modifying treatment. Current standard-of-care involves treatment of symptoms alongside multi-disciplinary support involving physicians, specialist nurses, therapists, clinical psychologists and dieticians to maintain independence and quality of life for as long as possible. Although HD is rare it has a prevalence of ∼1 in 8,000 in the UK^3^ making it likely that most physicians and general practitioners will need to manage HD patients during their careers, particularly given the high medical needs of this patient group. In this review we summarise aspects of the presentation, diagnosis and management of HD of most relevance to acute and general physicians.

## Genetic testing

HD is caused by an expanded repeat of at least 36 CAGs in one copy of the Huntingtin (*HTT*) gene.^4^ In unaffected individuals the tract has ∼18–20 CAGs (range 6–35^5^). Longer repeat lengths are associated with earlier onset and more severe disease, with CAG > 60 usually causing juvenile-onset HD before the age of 20.^6^ Genetic testing for HD, using a simple blood test to measure CAG length, is broadly carried out in two scenarios:

1) **Predictive testing in asymptomatic individuals at risk of developing HD**

Asymptomatic adults with a family history of HD can request predictive testing to determine if they carry the HD repeat expansion. Each child of an affected individual has a 50% chance of inheriting the HD mutation. In the UK, predictive testing is managed by Clinical Genetics services and usually involves multiple consultations to explore an individual's understanding of the test, as well as their support networks and strategies to cope with a positive test result. It is important to be aware that a positive predictive test for an individual can have implications for other family members who might have chosen not to undertake predictive testing. At present under 20% of asymptomatic at-risk individuals request a predictive test, principally because of the lack of a disease-modifying treatment.^7^ In addition, insurance companies are permitted to ask for disclosure of predictive HD test results if more than £500,000 of cover is requested. Predictive testing is not recommended for those under 18.

2) **Diagnostic testing in individuals with symptoms/signs that could be HD**

In individuals with symptoms and/or signs consistent with HD, most commonly chorea, a diagnostic HD genetic test might be offered. Mostly there will be a family history of HD, but ∼10% of positive diagnostic tests are apparently *‘de novo*’. Often an absence of family history will be due to adoption or estrangement, or misdiagnosis in earlier generations. For example, for an individual presenting with chorea, a strong family history of severe mental illness, institutionalisation, alcohol or drug dependence, ‘dementia’ or ‘Parkinson's disease’ can be suspicious for HD. Occasionally patients’ relatives have been diagnosed with ‘HD’ prior to the era of genetic testing. In this scenario HD genetic testing is useful as there are rare phenocopies of HD caused by other repeat expansions in genes such as *C9ORF72, TBP* and *ATN1*. Diagnostic HD testing in symptomatic individuals requires appropriate consent for a genetic test but not the same degree of counselling as for predictive testing. It is usually carried out by neurologists, clinical geneticists or psychiatrists.

## Presentation and investigation of HD

We will focus this primer on confirmed HD gene carriers. There are currently no NICE guidelines for investigation and management of HD and so recommendations are based on published expert consensus opinions.

### Presentation

HD affects many aspects of movement control, mental health, behaviour and cognition over its course (Table 1). Involuntary, unsuppressible choreiform (‘dance-like’) movements are characteristic of, but not specific to, HD and found in almost all patients at some stage. They usually start insidiously, fluctuate diurnally (reducing at night) and day-to-day, and can affect all muscle groups including those of the face, trunk and limbs. Chorea tends to progress slowly and the patient is often less troubled by the movements than care-givers. Other motor problems that can develop include dystonia, rigidity and incoordination, and these contribute to a significant falls risk that increases as the disease progresses. Later in the disease course, eye movements, speech and swallowing can all be affected leading to problems with communication and nutrition, and risk of aspiration. Reduced food intake and the catabolic state seen in HD lead to weight loss which also contributes to frailty and impaired physical abilities.

**Table 1:** Common clinical manifestations of Huntington's disease.

Domain	Clinical manifestation	Specific symptoms/signs commonly seen in HD	Medications that may be useful
Motor	Involuntary movements	Chorea	Tetrabenazine, 2nd generation anti-psychotics
		Dystonia	Botulinum toxin
	Bulbar dysfunction	Dysarthria	
		Dysphagia	
	Reduced balance and coordination	Eye movement problems	
		Rigidity	
		Ataxia	
Psychiatric	Depression	Blunted affect	SSRI, SNRI
		Suicidal ideation	SSRI, SNRI
	Anxiety disorders	Constant worry, nervousness	SSRI
		Irritability (short fuse)	SSRI (higher doses),
		Obsessive/compulsive phenomena	SSRI
	Psychosis	Delusions, paranoia	2nd generation anti-psychotics
		Hallucinations	2nd generation anti-psychotics
Behavioural	Apathy	Reduced motivation	
		Social withdrawal	
	Perseveration	Repetitive behaviours with little insight e.g. frequent toileting, hypersexual behaviours	SSRI, 2nd generation anti-psychotics
	Anger	Aggressive behaviours	2nd generation anti-psychotics
Cognitive	Inattention	Reduced concentration	
	Executive dysfunction	Slowed thinking, impaired planning	
	Poor memory	Disorientation	

Symptoms and signs that are frequently encountered in individuals with HD are divided across four major domains. Most patients will experience symptoms from each domain across their disease course, although exact combinations vary by individual. Medications that can be helpful are indicated, although there is very little clinical trial evidence to base decisions on. Key: SSRI, Serotonin-Selective Reuptake Inhibitor (SSRI); SNRI, Serotonin-Noradrenaline Reuptake Inhibitor (SNRI).

HD also causes significant psychiatric, behavioural and cognitive problems that directly affect an individual's ability to hold down a job, manage their finances, maintain close relationships and, ultimately, care for themselves. In over 40% of gene carriers these symptoms will predate any motor symptoms of HD.^5^ A mixture of neuropsychiatric symptoms is commonly reported by care-givers, with apathy, loss of motivation and perseverative thoughts and actions prominent. Depression, anxiety, irritability, aggressive outbursts and obsessive/compulsive thoughts and behaviours are also regularly observed, all exacerbated by disrupted sleep patterns. Less frequently, hallucinations and delusional behaviours are reported. Rates of substance misuse, self-harm and suicide are higher in the HD population than in the general population and rates of addiction often increase with disease progression.^8^

### Examination

Cranial nerve examination can highlight abnormal eye movements: jerky or incomplete ocular pursuit and/or slowed saccades. Dysarthria might be present as well as an inability to keep the tongue fully protruded. This ‘motor impersistence’ is a sign of chorea and may also be elicited through fluctuating grip strength when asked to grip the examiner's fingers (‘milkmaid sign’). Examination of the upper limbs can show incoordination of finger tapping and pronation/supination as well as a degree of rigidity. Observation of all body areas throughout a consultation can reveal chorea and dystonia, and these are often asymmetric, especially earlier in the disease course. Gait can be bradykinetic and stiff, and tandem walking difficult. As the disease progresses there is often loss of muscle mass, more widespread and unsuppressible involuntary movements, and progressive difficulty following commands in a standard examination. Cognitive testing often reveals impaired attention, executive function and memory.

### Investigations

**Fig. 1:**
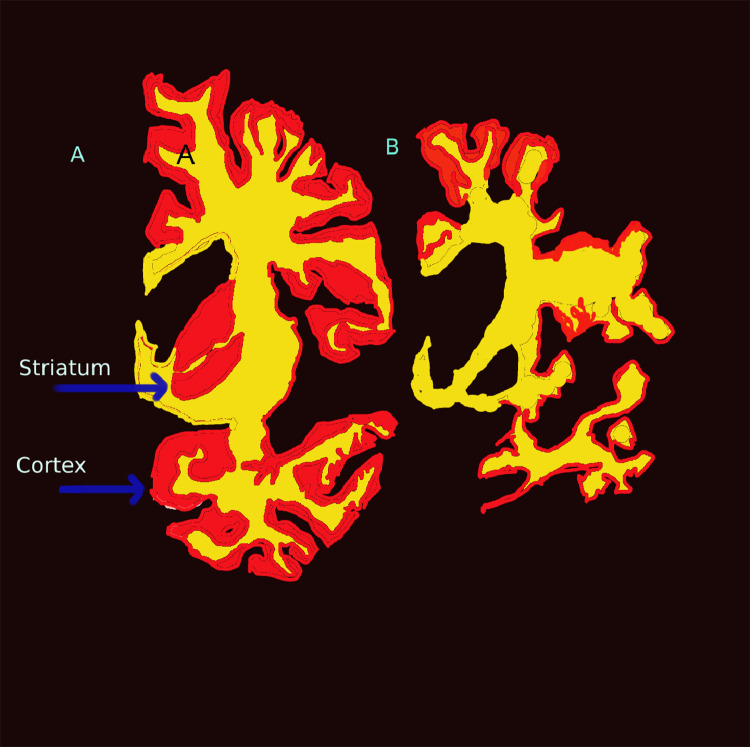
Progression of Huntington's disease from early motor stage (A) to late stage (B). The coronal sections through the left hemisphere demonstrate whole brain atrophy that is disproportionately focussed on the grey matter of the striatum and cortex (in red). The white matter (in yellow) is relatively preserved. There is almost complete loss of the striatum in later stages of the illness (B). Figure designed after Vonsattel, 1998.^10^

Beyond genetic testing, there are no specific investigations required for a diagnosis of HD. MRI or CT brain scanning can show caudate and then putaminal atrophy early in the disease course,^9^ with more widespread brain atrophy later on (Fig. 1).

## Acute presentations in individuals with HD

Progression of HD is usually slow, occurring over years. Therefore, acute deteriorations in HD-related symptoms often indicate another problem. For example, intercurrent urinary tract, chest or other infections can exacerbate HD symptoms as can pain, constipation and urinary retention. In our experience, gastro-oesophageal reflux disease and poor dentition can also often cause symptomatic exacerbation in HD. Changes in work, home, social or financial circumstances, and drug or alcohol use or abuse can all contribute to an acute deterioration in mental or physical health. Importantly, individuals with HD often lack insight into their condition and may also not be able to communicate their symptoms clearly, particularly in advanced disease. They can perseverate on particular symptoms and so careful assessment is needed to prioritise those requiring treatment.

As well as exacerbation of existing symptoms, individuals with HD can develop new problems unrelated to HD. These can be difficult to identify if they affect the nervous system: for example, a TIA or stroke might cause acute neurological symptoms that are mistaken for HD. Seizures are not usually part of adult HD and so their occurrence should prompt a full work-up.

## Management of HD-related symptoms

There are currently no treatments that can slow down or prevent onset or progression of HD. Medical and non-medical interventions are aimed at symptom management and maintenance of quality of life.^11^ Ideally, HD patients are managed by a specialist multi-disciplinary team including doctors, specialist nurses and a range of therapists.

### Medical therapy (Table 1)

If chorea is intrusive, tetrabenazine three-times daily can be used to reduce its severity.^12^ This depletes central monoamines and should be used with caution in those with depression or parkinsonism. Alternative, off-licence, medications for chorea include second generation antipsychotics such as olanzapine or quetiapine. These are favoured in those with co-existing behavioural or psychotic symptoms, and in those where once-daily dosing is easier, but should be used cautiously in those with rigidity and bradykinesia. Anti-choreic drugs should ideally be started and managed by a specialist.

Depression and anxiety are common in HD. First-line treatments include selective serotonin reuptake inhibitors (SSRIs), such as citalopram or sertraline, and serotonin-noradrenaline reuptake inhibitors (SNRIs), such as venlafaxine or duloxetine. Mirtazapine can also be useful, particularly if sleep is problematic. Irritability can respond to SSRIs, but often requires higher doses. Agitation and aggressive outbursts, as well as delusions and hallucinations, can be treated with antipsychotics. Short-term benzodiazepines can be used in acute situations.^11^

### Non-medical therapies

Therapies are central to enabling individuals with HD to live independently and well for as long as possible. Physiotherapy can help with balance, coordination and mobility.^13^ Speech and language therapists are essential for monitoring and improving communication and swallowing. Dietitians and nutritionists can advise on suitable soft but calorific diets to help patients maintain body mass. In individual cases where dysphagia is particularly problematic in advance of other aspects of HD, percutaneous endoscopic gastrostomy (PEG) tube placement and feeding may be considered.^14^ Occupational therapists can help adapt a patient's home to enable them to live there longer; for example, various assistance aids are available to assist mobility and communication in those with involuntary movements and impaired cognition. Psychological therapies are very useful for managing behavioural and psychiatric symptoms but are unevenly available through the UK.

## Considerations in advanced HD

HD inevitably progresses after symptom onset, usually over 10–20 years. As such, early assistance with applying for benefits, liaising with the DVLA about driving, and considering lasting powers of attorney, for both health and financial matters, can be invaluable for patients. Advanced care plans can be useful, including the patient's wishes around refusing treatment including resuscitation, and appropriate documentation put in place in both hospital and community settings. As the disease becomes more advanced, cognitive impairment becomes increasingly problematic, alongside neuropsychiatric symptoms, lack of insight and severely impaired mobility. Eventually patients are unable to manage daily tasks. Living at home might become impossible and patients often have to move to care settings. Palliative care input can be invaluable in the final stages of life to ensure comfort and dignity.^15^ The leading causes of death in HD are pneumonia and suicide.^2^

## Conclusions

Individuals with HD require considerable medical and psychiatric support over the course of their lives. Careful assessment is needed to discriminate between symptoms directly caused by HD and those of intercurrent illnesses. Although HD is progressive and there are no disease-modifying treatments, many acute deteriorations are reversible, particularly early in the disease course, with a combination of medications and therapist support. Pioneering treatments that lower mutant huntingtin protein levels or prevent CAG repeat expansions in the brain are currently in development, and there is optimism that a drug that slows or prevents onset of HD will be available in the next 5–10 years.^16^

## Take-home messages

• Acute deterioration in HD patients usually indicates acute illness.
• Gastro-oesophageal reflux disease and impaired dentition are common mimics of HD ‘progression’.
• Chorea is often not troublesome for patients, and may not need medication.
• Neuropsychiatric symptoms such as depression and irritability cause severe distress to patients and carers, but can respond well to SSRIs
• End-of-life care planning should begin early, with clear documentation linked to patient records

## Declaration of competing interest

T.H.M. is an associate member of the scientific advisory board of LoQus23 Therapeutics Ltd. D.J.M. reports no conflicts of interest.
